# Dental color assessment through TTB exercises

**Published:** 2016

**Authors:** R Draghici, CT Preoteasa, AMC Ţâncu, E Preoteasa

**Affiliations:** * Department of Prosthodontics, Faculty of Dental Medicine, “Carol Davila” University of Medicine and Pharmacy, Bucharest, Romania; ** Department of Scientific Research Methodology and Ergonomics, Faculty of Dental Medicine, “Carol Davila” University of Medicine and Pharmacy, Bucharest, Romania

**Keywords:** tooth color, Tooth Training Box, visual color determination

## Abstract

Abstract

**Aim.**To verify the impact of taking the Toothguide Training Box (TTB) exercise in improving the individual ability to correctly determine dental color.

**Material and method.**A prospective study was conducted on the 5th year dental students. The participants were required to carry out 3 distinct steps: visual color determination for sample tabs out of the 3DMaster shade guide, the TTB exercise and another color determination after training completion.

**Results.**The sample included 60 students (19M/ 41F) with a mean age of 24, which made 360 color determinations of 6 shade tabs before and after TTB exercise. 32,5% (n=117) of the color determinations were incorrect in both moments, and the value was incorrectly determined just in 11% of them. Students found 3L1.5 and 3M2 colors as the hardest to determine. The results suggested that a single TTB training exercise did not have a high positive influence on the individual capacity to correctly determine the color for tabs out of the shade guide.

**Conclusions.**While there is no evidence of an immediate positive impact of the TTB exercise in evaluating and determining different color variations for sample key elements, through repeat exercises, the individual perception can be improved and thus the correct determination of dental color and its correct codification can be increased.

Abbreviations: TTB = Tooth Training Box, CIE = Commission internationale de l'éclairage, L = lightness, C = chroma, H = hue

## Introduction

The patient’s esthetic requirements regarding dental restorations are primarily directed towards the recreation of the natural aspect of the teeth. Dental color is considered the most important esthetic component and its determination is directly correlated with the visual experience and perception of all participants. The perception of color involves the processing of signals from the cone cells in the retina, determining color contrast in the striatum cortex and perception stabilization in temporal areas [**1**].

In the constant attempt to create a common language regarding the color determination, there are several existing systems. The tridimensional cylindrical system for color [**2**] or the Munsell system, in which color dimensions were perceptually separated in hue, chroma, value. In 1976, the International Commission on Illumination (CIE) defined the tridimensional color space that supports the color perception based on 3 parameters (red, green, blue) with L*, a*, b* axes. Recent studies have shown that color perception can be represented on a color sphere with 4 dimensions [**3**], with the 4th dimension, “darkness”, being harder to visualize. 

Color can be subjectively expressed (based on the visual response of the observer) or objectively (based on the physical properties of color as measured by instruments) [**4**]. The visual analysis of teeth color in comparison with a dental color key is the fastest and least expensive method, most frequently used in dentistry [**5**], but still a subjective method [**6**]. The practitioners’ ability to determine the color improves over time but can also be positively influenced through training and practice [**7**].

Color distribution in Vitapan’s 3D-Master shade guide is performed according to Munsell’s principles, thus becoming more structured than the Vita Classical key and achieves increased reliability for repeated measurements [**8**]. Some disadvantages reported on the subjective method for dental color determination include the following: the number of shades do not cover the complete space of natural teeth [**9**,**10**], shades are not systematically arranged in the color space [**11**,**12**], there are inconsistencies between professionals in color determination [**13**], results cannot be transformed in the CIEL*a*b* system [**14**], and no commercial shade guide available is identical to another [**15**]. Variables such as external light, age, experience, tiredness, physiological conditions, medication, or eye diseases can lead to inconclusive results or errors. Despite such limitations, the human eye is highly sensitive in detecting tiny color differences between 2 objects [**16**].

The aim of this study was to check the extent to which undertaking the Tooth Training Box (TTB) exercise could contribute to the improvement of the individual capacity to correctly determine the dental color. 

## Methods

A prospective study was conducted on a sample group of 5th year students from the Dental Faculty of “Carol Davila” University of Medicine and Pharmacy in Bucharest. Students were selected on a volunteering base of participants to an optional course on Dental Esthetics. During the course assignment and seminars, students received basic notions on dental esthetics but also fundamental information about dental color parameters and determination methods using color keys. 

The study implied that the participants accomplished 3 distinct steps:

1. In step 1, time 1 (T1), participants filled out a worksheet that included personal details, age, and their determination of color for 6 tabs out of the 3D-Master shade guide. 

2. Subsequently, each student participated in a dental color determination exercise using the TTB device provided by Vita company as a part of the contract signed between “Carol Davila” University of Medicine and Pharmacy and the company. 

3. After the TTB exercise and the recording of the results for each student, they were asked to determine the color for the same 6 tabs out of the 3D-Master shade guide used in step 1 again and their determination time was measured (T2). 

For the purpose of this study, 6 tabs from the 3D-Master shade guide were selected (VITA Zahnfabrik, Bad Säckingen, Germany): 2R2,5; 3L1,5; 4R1,5; 3M2; 2M3; 4L1,5. The tabs were chosen together with a dental ceramic technician in the attempt to select colors that are hard to reproduce (4L1,5) or that are easy to confuse one with another (2M3-3M2). The 6 tabs were removed from the shade guide, the metal holder being kept for support. The color parameters written on the metal holder were covered with opaque adhesive tape that made it impossible for the written letters and numbers to be seen. The 6 tabs were randomly assigned values from 1 to 6 (**Table 1**).

**Table 1:**
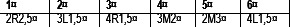
**Table 1**The distribution of the 6 shade tabs

The study took place between 12:00 and 14:00, the optimal time for color determination under natural light conditions. Students were assigned in groups of 8 and, prior to the exercise, were administered the Ishihara color vision test which is useful to check for color sight in larger groups of people [**17**].

For step 2, students received instructions regarding the device, its functioning, and the training steps in order to participate in the Tooth Training Box (TTB) exercise. TTB is a device, which was invented in 2002, by Prof. Dr. H.A. Jakstadt from Leipzig University and manufactured by Norwark Enginering. It includes an electro mechanic device connected to a software system. The 3D Master shade guide is horizontally embedded on the lower part of the device, while in the upper part, 52 teeth are radially arranged. As a part of the exercise, one of the 52 teeth is automatically selected and the participant has to correctly select the parameters from the horizontal shade guide. The visualization of the test tooth and the selected group from the color key is performed through an opening (60x60 mm) placed in the center of the mechanical device. The parameter selection is performed through arrow buttons that position the key in accordance with the participant selection. Training involves undergoing 3 practice exercises with varying levels of difficulty and the final test that measures knowledge and color selection capacity through a final assigned score. In the first practice exercise, the subject needs to correctly select the lightness of the test tooth, while in the second, both lightness and chroma need to be determined. In exercise 3, the participant needs to select the appropriate corresponding tooth from the color key by correctly appreciating all 3 parameters: lightness, chroma and hue. Each exercise requires the correct determination for 8 teeth tests, otherwise the participants are not able to move forward until a correct determination is made. After the completion of the 3 practice exercises, participants need to undergo a final test in which all three color parameters need to be determined for 15 test teeth. At the end of the test, they receive a score with a maximum of 1000 points. While each parameter is important in dental color determination, the lightness parameter has a greater weight in the assigned score. 

In step 3 of our study, the same 6 tabs from the 3D-Master shade guide, whose color was initially determined, were re-assessed by the participants and the results were recorded on a different worksheet. All assessments were individual and they were carried out under the same environmental conditions. 

## Results

The sample group consisted of 60 students, 19 males, and 41 females, aged between 22 and 50 years, with an average age of 24. Each student performed the color determination for 6 tabs out of the shade guide (before and after the TTB exercise), a total of 360 color determinations at two points in time. 

Improving the ability for dental color determination is a desirable goal, given that an important percentage of the evaluations (n=117; 32,5%) were incorrect in both assessments (T1 and T2). One can observe that value (L) – the color component perceived as having the highest weight in visual perception – was incorrectly determined at both moments in time (before and after) in just 40 cases out of the 360 (11%). Analyzing the correctness of color determination for the 6 tabs from the 3D-Master shade guide prior and after the TTB exercise, it was observed that in general, there was no impact on the assessment of the 6 tabs from the key or even a negative impact as in the case of value determination (**Table 2**). 

**Table 2:**
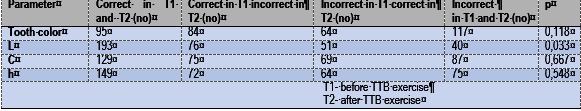
**Table 2**Accuracy in the determination of dental color components

Students perceived the teeth colored 3L1,5 and 3M2 as the hardest to evaluate (**Table 3**).

**Table 3:**
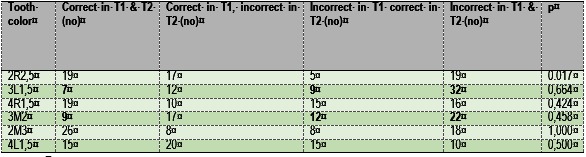
**Table 3**Accuracy in dental color determination for the 6 tabs

Students who failed to correctly determine the color of one of the teeth during the allocated time in the TTB exercise received lower scores than those who managed at least once, before or prior to the TTB exercise (**Table 4**).

**Table 4:**
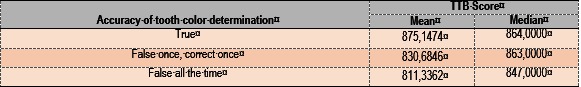
**Table 4**TTB score distribution according to the accuracy of the determination

Using the Kruskal-Wallis test, it was observed that there were statistical significant differences in the scores of the students who have always correctly determined the color, those who have determined it at least once and those who have never managed a correct identification (p<0.0001). Using the Mann-Whitney test, it was observed that there were no differences between those students who have always correctly determined color and those who have determined it correctly at least once but there were differences between the latter ones and those students who have never correctly determined color at any point in time (p<0,001 and p=0,001).

## Conclusions

Dental color determination is an important topic for dental practitioners especially for those involved in dental restorations, in which, in order to meet esthetic requirements, color must be correctly identified and used in relation with all the oral structures. To this point, dental color determination exercises have been able to illustrate the capacity to perceive dental color for all individuals involved in a case, have been able to train regarding the understanding of the complexity of the dental color as well as reveal practical aspects related to the determination and use of dental restorations in medical procedures.

Our study showed that, in the case of dental color determination exercise, an important percentage of incorrect evaluations were for chroma (c) and hue (h), while a somewhat lower value (L) was expressed for the parameter of color, considered as having the highest impact in the visual perception regarding the tooth color determination. 

Improving one’s ability to determine color is an important and necessary aspect for dental restorations, especially for young professionals, but also for those who have difficulties in perceiving colors. Our study showed that individuals who have always failed to correctly determine color during the exercise with tabs from the 3D-Master shade guide have consistently recorded low scores on the TTB exercise. 

While no immediate positive impact was observed after one TTB exercise, associated with the identification and determination of shades tabs out of the shade guide, through repeated exercises, individual perception could be improved, thus increasing the accuracy in the determination and use of dental color, in prosthetic restorations. 

### Acknowledgement

Doctoral and postdoctoral research priority of the Romanian higher education (Doc-Postdoc) POSDRU/159/1.5/S/137390.

### Disclosures

None

## References

[1] Barton J (2014). Visual color and form perception. Encyclopedia of the Neurological Sciences Elsevier Ltd..

[2] Barton J (2008). Forgotten pioneers of colour order. Part II: Mattias Klotz (1748-1821). Color Research and Application.

[3] Leonov Y, Sokolov E (2008). The representation of colors in spherical space. Color Research and Application.

[4] Hunt R (1977). The specification of colour appearances: concepts and terms. Color Research and Applications.

[5] Van der Burgt T, Ten Bosch JJ, Borsboom Pand, Kortsmit W (1990). A comparison of new and conventional methods for quantification of tooth color. Journal of Prosthetic Dentistry.

[6] Imbery T, Geissberger M, Hakim F, Al-Anezi S, Uram-Tuculescu S, Gottlieb R, Estrich G (2013). Evaluation of four dental clinical spectrophotometers relative to human shade observation. Journal of the American Dental Association.

[7] Al-Dosari A (2010). Reliability of tooth shade perception by dental professionals and patients. Pakistan Oral and Dental Journal.

[8] Hammad I (2013). Intrarater repeatability of shade selections with two shade guides. Journal of Prosthetic Dentistry.

[9] Schwabacher GR (1990). Three-dimensional color coordinates of natural teeth compared with three shade guides. Journal of Prosthetic Dentistry.

[10] Hall N (1991). Tooth colour selection: the application of colour science to dental colour matching. Australian Prosthodontic Journal.

[11] Sproull R (1973). Color matching in dentistry. Part II. Practical applications for the organisation of color. Journal of Prosthetic Dentistry.

[12] O’Brien W, Groh C, Boenke K (1990). A new, small-colordifference equation for dental shades. Journal of Dental Research.

[13] Okubo S, Kanawati A, Richards M, Childress S (1998). Evaluation of visual and instrument shade matching. Journal of Prosthetic Dentistry.

[14] Donahue J, Goodkind R, Schwabacher W, Aeppli D (1991). Shade color discrimination by men and women. Journal of Prosthetic Dentistry.

[15] Yap A (1998). Color attributes and accuracy of vita-based manufacturers’ shade guides. Operative Dentistry 1.

[16] Paul S, Peter A, Pietroban N, Hammerle C (2002). Visual and spectrophotometric shade analysis of human teeth. Journal of Dental Research.

[17] Bratner S, Vichi A, Borbely J, Jakstat H (2010). The Ishihara test as a data-projection-still a valid screening tool to test redgreen color deficiency. Deutsche Zahnarztliche Zeitschrift.

[18] Robertson A (1978). Recent CIE work on color-difference evaluation. Color Research and Applications.

[19] Chu S, Devigus A, Paravina R, Mieleszko A (2010). Fundamentals of Color: Shade matching in Esthetic Dentistry.Quintessence Books.

[20] Joiner A (2004). Tooth colour: a review of the literature. Journal of Dentistry.

[21] Wunnemann P (2009). Tooth colour: a review of the literature. Med Diss..

[22] Olms C, Klinke T, Pirek P, Hannak W (2013). Randomized multi-centre study on the effect of training on tooth shade matching. Journal of Dentistry.

[23] Hannak W (2006). Can the ability to identify tooth color differences be learned?. 84th General Session & Exhibition of the IADR 1st Meeting of the Pan-Asian-Pacific Federation Brisbane.

[24] Xu M, Xu T, Liu F, Shi X, Feng H (2009). The influence of toothguide training box on shade matching veracity. Shanghai Journal of Stomatology.

[25] Virdee P, Louca C, Tredwin C (2010). Investigation of the Training Benefits of the Toothguide Training Box. IADR General Session.

[26] Llena C, Forner L, Ferrari M, Amengual J, Llambes G, Lozano E (2011). Toothguide Training Box for dental color choice training. Journal of Dental Education.

